# Cardiac medication use in patients with suspected ischaemia without obstructive coronary arteries: sex differences and psychological distress

**DOI:** 10.1007/s12471-021-01569-4

**Published:** 2021-05-05

**Authors:** P. M. C. Mommersteeg, J. Roeters van Lennep, J. Widdershoven

**Affiliations:** 1 Department of Medical and Clinical Psychology, Centre of Research on Psychology in Somatic Diseases, Tilburg University, Tilburg, The Netherlands; 2 Department of Internal Medicine, Vascular Medicine, Erasmus Medical Centre, Rotterdam, The Netherlands; 3 Department of Cardiology, Elisabeth-TweeSteden Hospital, Tilburg, The Netherlands

**Keywords:** Ischaemia without obstructive coronary arteries, Ischaemic heart disease, Sex differences, Medication use, Depression, Anxiety

## Abstract

**Background:**

Ischaemia without obstructive coronary arteries (INOCA) is more prevalent in women and associated with psychological distress. Pharmacological treatment goals are angina relief and cardiovascular risk management. The present study aims to examine sex differences in cardiac and non-cardiac medication use, as well as medication and sex differences related to consistent psychological distress in patients with suspected INOCA.

**Design:**

A TweeSteden mild stenosis observational cohort study in patients with suspected INOCA as detected by ischaemic reason for referral and non-obstructive arteries based on coronary angiography or computed tomography.

**Methods:**

Medication documented in the hospital records of 488 patients (53% women) was coded as angina relief medication, blood-pressure-lowering medication, antithrombotics, statins, and non-cardiac medication, using the Anatomical Therapeutic Chemical code. Depressive symptoms and anxiety were recoded as ‘consistent distress’ (above the cut-off score for depression and anxiety on validated questionnaires), ‘inconsistent distress’ (above the cut-off for depression or anxiety) or ‘no distress’ (below the cut-off).

**Results:**

No sex differences were observed in cardiac medication use. Women used anxiolytic benzodiazepines more often (12% vs 4%, *p* = 0.002) compared to men. Consistent distress was more prevalent in women (22% vs 15%, *p* = 0.004) and was related to the use of more angiotensin-converting enzyme inhibitors/angiotensin receptor blockers and diuretics in women and to calcium antagonist use as well as lower adherence levels in men. Women who reported chest pain more often received angina relief medication and blood-pressure-lowering medication than men.

**Conclusion:**

No sex differences were observed in cardiac medication use in patients with suspected INOCA. Psychological distress may reflect hypertension and subsequent medication use in women, and experiencing chest pain and subsequent medication use in men.

## What’s new?

- No sex differences were found in cardiac medication use in patients with suspected ischaemia without obstructive coronary arteries.
- Consistent psychological distress was related to the use of more angiotensin-converting enzyme inhibitors/angiotensin receptor blockers as well as diuretics in women.
- Psychological distress was related to more calcium antagonist use, as well as to lower medication adherence in men.

## Introduction

Ischaemic heart disease (IHD) is the leading cause of death worldwide [1]. IHD without significant obstruction of the coronary arteries is referred to as non-obstructive coronary artery disease or, in the presence of signs and symptoms of ischaemia, ischaemia without obstructive coronary arteries (INOCA) [2]. Obstructive IHD is more prevalent in men, whereas INOCA is more prevalent in women [3]. Treatment for patients with IHD is based upon the European Society of Cardiology guidelines, consisting of managing cardiovascular risk factors by a healthy lifestyle and pharmacological therapy in combination with patient education [4, 5].

Psychosocial factors such as depression, anxiety and experiencing high levels of both anxiety and depressive symptoms indicating psychological distress are highly prevalent in IHD patients [6], particularly in women [7]. Psychological distress, depression, and anxiety are known to adversely affect medication adherence [8] and prognosis [9], for which appropriate referral is beneficial to reduce psychological symptoms [6, 10]. In the present study, sex differences in the use of cardiac and non-cardiac medications and in psychological distress were examined in patients with suspected INOCA.

In patients with IHD, pharmacological therapy aims to provide relief for angina-like symptoms and to prevent future cardiac events [4, 5]. A study comparing medical treatment between women and men among > 52,000 Dutch patients after a myocardial infarction showed that women were less likely to receive optimal medication treatment, as indicated by the presence of acetylsalicylic acid, P2Y12 inhibitor, statins, beta blockers and angiotensin-converting enzyme/angiotensin 2 inhibitors [11]. It is unclear if these differences are present in patients with suspected INOCA. Women and men display differences in pharmacodynamics and pharmacokinetics, and women more often report more side effects [12]. Moreover, women with INOCA were more likely to be readmitted than men [3]. Still, women diagnosed with INOCA experience a two-fold increased risk for obstructive coronary artery disease (CAD) in the next 5–8 years and a four-fold increased risk for (cardiovascular) hospitalisation [13]. However, the absence of a sex-sensitive risk for adverse outcomes has been observed [14], and mortality rates were found to be similar for women and men with INOCA [15, 16]. It is unknown if medication use is different for women and men with INOCA, and if these differences are apparent in women and men who report psychological distress.

We aim to add to this framework by examining sex differences in the medical treatment of patients with suspected INOCA. Suspected INOCA is defined as having visible wall irregularities or mild stenosis based on consecutive routine coronary angiography (CAG) or computed tomography (CT) scans in patients referred for cardiac complaints suspicious of ischaemia. We hypothesise that women receive cardiac medication, including angina relief medication, as well as preventive cardiovascular medication such as anticoagulants/antiplatelets, statins, or blood-pressure-lowering drugs, less often than men. Second, we aim to examine the sex-stratified effect of psychological ‘consistent distress’, defined as experiencing high levels of both anxiety and depressive symptoms, on medication use and medication adherence. Given a higher symptom burden in patients with depressive symptoms or anxiety, we hypothesise that consistent psychological distress is related to more medication treatment, but lower adherence, for which sex differences may be apparent.

## Materials and methods

### Participants and procedure

The present study is a secondary analysis of the TweeSteden Mild Stenosis (TWIST) study [17]. Consecutive patients undergoing CAG (*n* = 5638) or 64-slice CT scan (*n* = 852) were screened between January 2009 and February 2013 [17]. All patients received treatment as usual from their cardiologist. Inclusion criteria were a CT calcium score above 0 without undergoing CAG, or CAG-detected visible mild stenosis (< 50% left main coronary artery; < 70% other arteries). Exclusion criteria were absence of visible irregularities (≤ 20%), significant coronary stenosis or a history of cardiac events. Eligible patients (*n* = 883) received information about the study, and 547 patients (62%) gave their signed informed consent. Data were collected within 3 months after CAG or CT scan, and questionnaires were sent and returned by postal mail. Hospital record information on the reason for referral for CAG or CT scan was categorised to examine ischaemic [*n* = 209, 38%; (unstable) angina pectoris; acute coronary syndrome; ischaemia based on electrocardiography, ergometry, or myocardial perfusion imaging] and suspected ischaemic origins (*n* = 238, 44%; inconclusive ergometry or myocardial perfusion imaging test; atypical chest pain). Patients referred for having a high number of risk factors, a high familial risk or other non-ischaemic reasons were excluded from the present analysis (*n* = 99, 18%), leaving 448 patients with suspected INOCA. The research protocol was approved by the Medical Ethics Committee (METC Brabant; NL22258.008.08) and registered as an observational cohort study at ClinicalTrials.gov (NCT01788241).

### Medication and adherence

Pharmacological products listed in hospital records were recoded as present or absent according to the Anatomical Therapeutic Chemical (ATC) code [18]. Treatment for patients without obstructive CAD aims to provide relief for angina-like symptoms using nitrate vasodilators, beta blockers or calcium antagonists, and to prevent future cardiac events by lowering blood pressure, taking antithrombotic medication and/or lipid-lowering drugs [4, 5]. Vasodilators included nitrate vasodilators (ATC code: C01DAxx): either short acting (C01DA02) or long acting (C01DA08, C01DA14). Blood-pressure-lowering medication included angiotensin-converting enzyme inhibitor (ACEI)/angiotensin II receptor blocker (ARB) [C09xxxx; angiotensin-converting enzyme inhibitor or an angiotensin II receptor type 1 antagonist (angiotensin II receptor blocker)], beta blockers (C07xxxx; C07AAxx, C07ABxx, C07BBxx), calcium antagonists [C08xxxx; C08DAxx, C08Dxx, with subgroup diltiazem (Tildiem; C08DB01)] and diuretics (C03xxxx, C07BBxx, C09BAxx, C09DAxx). Blood pressure medication was any of the above blood-pressure-lowering medications. Antithrombotic drugs included anticoagulants and antiplatelet drugs. Platelet inhibitors were B01ACxx; acetylsalicylic acid, or dipyridamole (B01AC07, B01AC30) and subgroups acetylsalicylic acid (B01AC06 aspirin or ASA), P2Y12 inhibitors (B01AC04 clopidogrel, B01AC24 ticagrelor, or B01AC30), and vitamin K antagonists (coumarins B01AA07 or B01AA04). No new oral anticoagulants were reported. Lipid-lowering medications were statins (C10xxxx; C10AAxx, C10ABxx, C10AXxx, C10BAxx, C10AX09).

Psychotropic drugs were grouped into antidepressants (N06AAxx, N06ABxx,) and sleep or anxiolytic benzodiazepines (N05CFxx/NO5CDxx, N05BAxx). Other non-cardiac medication included medication for diabetes (A10xxxx), chronic obstructive pulmonary disease (R03xxxx, R06xxxx), thyroid disease (H03AA01, H03BB02), proton pump inhibitors (A02BCxx), and non-steroidal anti-inflammatory drugs (M01Axxx).

In the questionnaire at 12 and 24 months patients were asked how often in the past month they had adhered to their medication treatment as prescribed by their physician, which was recoded as ‘always’ or ‘not always’.

### Psychological distress

Depressive symptoms and anxiety were reported using validated questionnaires, including the Beck Depression Inventory (BDI) for depressive symptoms and the Hospital Anxiety and Depression Scale for anxiety and depressive symptoms (HADS). The BDI has 21 items scored on a scale of 0–3, with a cut-off of ≥ 10 for moderate/severe depression. The HADS consists of two 7‑item scales measuring anxiety (HADS-A) and depressive symptoms (HADS-D), with a score range of 0–21 and a cut-off score ≥ 8 for moderate/severe symptoms. Scoring above the cut-off value on each questionnaire was coded as ‘consistent distress’. Scoring above the cut-off on one or two questionnaires was coded as ‘inconsistent distress’, and ‘no distress’ was the absence of values above the cut-off.

### Cardiac and non-cardiac risk factors

Sociodemographic factors included sex, age, having a partner, having at least college education and lifestyle risk factors: body mass index (BMI) and obesity (BMI ≥ 30), smoking (‘current smoker’ versus ‘former smoker’ or ‘never smoked’) and physical activity (being physically ‘active’ versus ‘inactive’ or ‘moderately active’). Cardiac risk factors were obtained from hospital records: first-degree familial heart disease before 60 years, hypertension, hypercholesterolaemia, diabetes, and comorbid conditions. Disease severity was coded as inclusion via CAG versus CT scan, since CAG is related to a more severe disease presentation [19]. The number of coronary arteries with wall irregularities was reported. Chest pain was coded as part of the modified version of the Seattle Angina questionnaire as having had any angina, chest pain or chest tightness in the past 4 weeks [17].

### Statistics

Analyses were completed with SPSS version 24. Sex-stratified and/or distress-stratified differences were examined using one-way ANOVA for continuous variables and Pearson chi-square tests for categorical variables. In cases with a low number per cell, Fisher’s exact test was used. Effect size phi-coefficient [φ = SQRT(χ^2^/*N*)] was calculated, for which φ 0.1 = small, φ 0.3 = medium and φ 0.5 = large. For descriptive purposes the prevalence of having medication in each category for having a specific risk factor was displayed for women and men, using chi-square tests to examine sex differences.

## Results

Consistent distress was more prevalent in women (22%) compared to men (15%, χ^2^ = 11.0, *p* = 0.004, effect size φ = 0.16) (Tab. 1). Moreover, women were older, less often had a partner, and received less college education than men. Of the patients with suspected INOCA, 67% had antithrombotic medication, statins (60%), blood-pressure-lowering therapy (67%) or angina relief medication (62%); there were no significant sex differences (Tab. 2). Women used benzodiazepines (12%) and thyroid medication (15%) more often than men (4% and 2% respectively), with small-medium effect sizes (Tab. 2, range φ = 0.15–0.22). Medication adherence at 12 months was not different between women and men, whereas more women (87%) than men (77%) reported always being adherent at 24 months (χ^2^ = 5.7, *p* = 0.017, φ = 0.11).

**Table 1:** Descriptive factors of patients with suspected ischaemia without obstructive coronary arteries, stratified by sex

		Women (53%, *N* = 239)	Men (47%, *N* = 209)		
	*N*	% (*n*) or mean (SD)	% (*n*) or mean (SD)	*χ*^2^*/F*	*p-*value
*Sociodemographic factors*					
Age (years)	448	63.32 (9.04)	60.51 (9.60)	10.19	0.002
With partner	427	73% (169)	90% (178)	19.85	< 0.001
College education or higher	424	43% (98)	71% (140)	33.33	< 0.001
*Lifestyle risk factors*					
BMI (kg/m^2^)	440	27.90 (4.66)	27.35 (3.44)	1.92	0.166
Obesity (BMI ≥ 30)	440	29% (69)	21% (43)	3.84	0.050
Smoking	446	18% (43)	21% (43)	0.48	0.487
Physically active	427	67% (154)	58% (114)	3.75	0.053
*Cardiac risk factors*					
Familial heart disease <60 years	421	68% (154)	59% (116)	3.41	0.065
Hypertension	444	85% (201)	84% (174)	0.05	0.827
Hypercholesterolaemia	446	67% (159)	70% (146)	0.59	0.443
Diabetes mellitus	445	14% (32)	13% (26)	0.10	0.754
*Reason for CAG/CT referral*					
Ischaemic^a^	239	54% (128)	53% (111)	0.01	0.925
Suspected ischaemic^b^	209	46% (111)	47% (98)		
*Disease severity*					
Diagnosis via CAG (vs CT scan)	448	73% (175)	76% (159)	0.48	0.489
Calcium score (mean % in CT group)	114	70.63 (19.34)	57.00 (19.14)	14.05	< 0.001
Visible wall irregularities	448				
One vessel	101	28% (68)	16% (33)	10.77	0.005
Two vessels	233	49% (118)	55% (115)		
Three or more vessels	114	22% (53)	29% (61)		
Chest pain in the past month	428	45% (103)	52% (103)	2.23	0.135
*Comorbid conditions*					
Peripheral artery disease	445	5% (12)	6% (13)	0.29	0.588
History of TIA or stroke	445	4% (9)	4% (8)	0.00	0.979
COPD	445	18% (42)	11% (23)	3.94	0.047
Inflammatory condition	445	11% (25)	7% (15)	1.51	0.219
Gastro-intestinal condition	445	16% (38)	13% (26)	1.12	0.289
Thyroid condition	445	18% (42)	2% (5)	27.52	< 0.001
Skeletomuscular conditions	445	9% (22)	10% (21)	0.08	0.772
Psychiatric condition	445	6% (15)	3% (6)	2.92	0.087
*Psychological distress*					
BDI depressive symptoms	426	10.76 (7.39)	8.71 (7.51)	8.09	0.005
High BDI score ≥10	426	48% (111)	32% (62)	12.13	< 0.001
HADS depressive symptoms	427	5.45 (3.98)	5.19 (4.19)	0.44	0.509
High HADS‑D score ≥ 8	427	29% (66)	26% (52)	0.35	0.556
HADS anxiety	427	7.13 (4.31)	5.96 (4.29)	7.86	0.005
High HADS‑A score ≥ 8	427	45% (103)	32% (63)	7.74	0.005
*Psychological distress*^c^					
Consistent distress (all scores high)	428	22% (51)	15% (30)	11.01	0.004
Inconsistent distress		39% (89)	30% (59)		
No distress (all scores low)		39% (90)	55% (109)		

*BDI* Beck Depression Inventory, *BMI* body mass index, *CAG* coronary angiography, *COPD* chronic obstructive pulmonary disease, *CT* computed tomography, *HADS* Hospital Anxiety and Depression Scale, *TIA* transient ischaemic attack

^a^Ischaemic = (unstable) angina pectoris; acute coronary syndrome; ischaemia based on ECG, ergometry, or myocardial perfusion imaging

^b^Suspected ischaemic = having an inconclusive ergometry or myocardial perfusion imaging test; atypical chest pain

^c^Psychological distress is operationalised as the presence of a high level of depression and anxiety according to the cut-off scores for the HADS‑D, HADS‑A, and BDI (consistent distress), or scores above the cut-off on one or two questionnaires (inconsistent distress), versus no score above the cut-off (no distress)

**Table 2:** Cardiac and non-cardiac medication stratified by sex

	Women (*N* = 237)	Men (*N* = 207)	Test value	*p-*value
	% (*n*)	% (*n*)	*χ*^*2*^	*p*
**Cardiac medication**				
Antithrombotic	64% (152)	70% (145)	1.75	0.187
– Platelet inhibitors (B01AC)	62% (148)	70% (145)	2.84	0.092
– Acetylsalicylic acid (B01AC06)	59% (141)	67% (139)	2.78	0.095
– P2Y12 inhibitor (B01AC04, B01AC24)^a^	5% (12)	4% (8)	0.37	0.544
– Vitamin K antagonist (B01AA)^a^	3% (7)	3% (6)	0.00	0.973
Statins (C10)	57% (135)	64% (133)	2.45	0.117
Blood-pressure-lowering medication	69% (163)	65% (135)	0.63	0.426
– ACEI/ARB (C09)	29% (68)	29% (60)	0.01	0.946
– Beta blockers (C07)	47% (112)	47% (98)	0.00	0.986
– Calcium antagonists (C08)	19% (44)	17% (36)	0.10	0.748
– Diltiazem (C08DB01)	7% (16)	6% (12)	0.17	0.680
– Diuretics (C03/C07BB/C09)	25% (59)	18% (37)	3.21	0.073
**Use of antithrombotics, statins, or blood-pressure-lowering medication**				
– None	9% (22)	11% (23)	4.51	0.212
– One	22% (53)	15% (32)		
– Two	38% (89)	36% (75)		
– Three	31% (73)	37% (77)		
**Nitrates (C01DA)**	18% (42)	16% (34)	0.13	0.718
– Short-acting nitrates (C01DA02)	11% (27)	14% (29)	0.69	0.407
– Long-acting nitrates (C01DA08, C01DA14)	11% (26)	6% (13)	3.03	0.082
**Angina relief medication**^**b**^	63% (149)	60% (124)	0.41	0.522
**Non-cardiac medication**				
*Psychotropic medication*				
– Antidepressant use (N06A)	10% (24)	6% (12)	2.78	0.095
– Benzodiazepine use (N05)^a^	12% (28)	4% (8)	9.37	0.002
*Other medication*				
– Diabetes medication (A10)	10% (24)	11% (22)	0.03	0.863
– COPD medication (R03)	13% (31)	12% (24)	0.23	0.635
– NSAIDs (M01A)^a^	8% (18)	3% (7)	3.69	0.055
– Proton pump-inhibitors (A02BC)	32% (76)	25% (52)	2.60	0.107
– Thyroid medication (H03AA01, H03BB02)^a^	15% (35)	2% (5)	20.57	< 0.001
**Medication adherence** (*N* = 401)				
– Always adherent at 12 months (vs not always)	83% (139)	78% (124)	1.42	0.234
– Always adherent at 24 months	87% (150)	77% (126)	5.66	0.017

*ACEI* angiotensin-converting enzyme inhibitor,* ARB* angiotensin II receptor blocker, *COPD* chronic obstructive pulmonary disease, *NSAIDs* non-steroidal anti-inflammatory drugs

^a^Similar findings using the Fisher exact test

^b^Angina relief medication was use of either nitrate vasodilators, beta blockers, or calcium antagonists

Tab. 3 shows that in women with consistent distress, significantly more ACEI/ARB were used (43%) than by women with inconsistent distress (28%), and no distress (22%)(φ = 0.17). A similar pattern was observed for the use of diuretics (41%, 25%, 18%; φ = 0.20). In men, the consistent distress group more often used calcium antagonists (33%, 19%, 13%; φ = 0.18) and proton pump inhibitors (43%, 17%, 23%%; φ = 0.20). Antidepressant and benzodiazepine use was more prevalent in the consistent distress group in both women and men (range φ = 0.19–0.37). Distressed men were less often ‘always adherent’, but only at 12 months (55%, 77%, 84%; φ = 0.19). An explorative analysis showed that women with consistent distress, as compared to inconsistent or no distress, had hypertension significantly more often (67%, 47%, 45%, φ = 0.18, data not shown), whereas men with consistent distress more often reported chest pain (83%, 58%, 40% respectively, φ = 0.31, not shown).

**Table 3:** Cardiac and non-cardiac medication use stratified by sex and distress

	Women *N* = 229 (54%)				Men *N* = 197 (46%)			
	Consistent distress	Inconsistent distress	No distress	Test	Consistent distress	Inconsistent distress	No distress	Test
	22% (51)	39% (89)	39% (89)		15% (30)	30% (59)	55% (108)	11.05**
**Cardiac medication**								
Antithrombotic	67% (34)	60% (53)	66% (59)	1.12	63% (19)	76% (45)	69% (75)	1.75
Platelet inhibitors (B01AC)	67% (34)	60% (53)	58% (52)	1.00	63% (19)	76% (45)	64% (69)	2.95
Statins (C10)	55% (28)	56% (50)	58% (52)	0.19	67% (20)	69% (41)	64% (69)	0.54
Blood-pressure-lowering medication	73% (37)	67% (60)	69% (61)	0.41	73% (22)	68% (40)	64% (69)	1.00
– ACEI/ARB (C09)	43% (22)	28% (25)	22% (20)	6.79*	47% (14)	27% (16)	26% (28)	5.08
– Beta blockers (C07)	53% (27)	45% (40)	47% (42)	0.84	43% (13)	51% (30)	47% (51)	0.47
– Calcium antagonists (C08)	24% (12)	17% (15)	18% (16)	1.01	33% (10)	19% (11)	13% (14)	6.71*
– Diuretics (C03/C07BB/C09)	41% (21)	25% (22)	18% (16)	9.21**	30% (9)	19% (11)	16% (17)	3.13
**Use of antithrombotics, statins or blood-pressure-lowering medication**								
– None	10% (5)	10% (9)	9% (8)	2.15	7% (2)	7% (4)	14% (15)	4.67
– One	22% (11)	22% (20)	22% (20)		17% (5)	12% (7)	15% (16)	
– Two	33% (17)	42% (37)	35% (31)		43% (13)	42% (25)	31% (34)	
– Three	35% (18)	26% (23)	34% (30)		33% (10)	39% (23)	40% (43)	
**Nitrates (C01DA)**	22% (11)	12% (11)	21% (19)	3.05	17% (5)	24% (14)	12% (13)	3.84
**Angina relief medication**^**a**^	67% (34)	64% (57)	61% (54)	0.53	67% (20)	61% (36)	58% (63)	0.70
**Psychotropic medication**								
– Antidepressant (N06A)	18% (9)	13% (12)	3% (3)	8.44*	27% (8)	5% (3)	1% (1)	27.34***
– Benzodiazepine (N05)	25% (13)	9% (8)	7% (6)	12.06**	13% (4)	2% (1)	3% (3)	7.93*
**Other medication**								
– Diabetes medication (A10)	16% (8)	11% (10)	6% (5)	3.87	20% (6)	10% (6)	8% (9)	3.38
– COPD medication (R03)	22% (11)	9% (8)	13% (12)	4.38	20% (6)	14% (8)	9% (10)	2.68
– NSAIDs (M01A)	6% (3)	7% (6)	9% (8)	0.55	7% (2)	5% (3)	2% (2)	2.17
– Proton pump inhibitors (A02BC)	41% (21)	36% (32)	25% (22)	4.67	43% (13)	17% (10)	23% (25)	7.71*
– Thyroid medication (H03AA01, H03BB02)	20% (10)	16% (14)	12% (11)	1.34	0% (0)	7% (4)	1% (1)	6.21*
**Medication adherence**								
– Always adherent at 12 months	79% (31)	83% (52)	85% (56)	0.50	55% (12)	77% (40)	84% (72)	8.57*
– Always adherent at 24 months	86% (32)	88% (58)	87% (60)	0.05	68% (15)	80% (40)	78% (71)	1.28

% (*n*) are reported, with *χ*^2^ test values

Distress is defined as the presence of high levels of depression and anxiety according to the cut-offs for the HADS‑D, HADS‑A, and BDI (consistent distress), or scores above the cut-off on one or two questionnaires (inconsistent distress), versus no scores above the cut-off (no distress)

*ACEI* angiotensin-converting enzyme inhibitor, *ARB* angiotensin II receptor blocker, *COPD* chronic obstructive pulmonary disease, *NSAIDs* non-steroidal anti-inflammatory drugs, *HADS* Hospital Anxiety and Depression Scale, *BDI* Beck Depression Inventory

*p < 0.01, **p < 0.05, ***p < 0.001

^a^Angina relief medication was use of either nitrate vasodilators, beta blockers or calcium antagonists

**Fig. 1:**
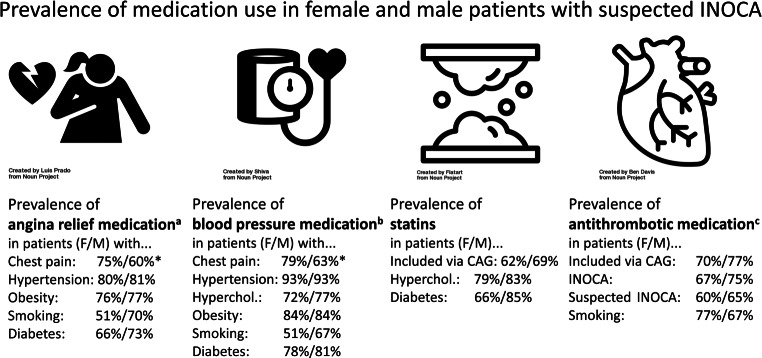
Sex-stratified prevalence of cardiac medication use in patients with suspected ischaemia without obstructive coronary arteries (*INOCA*). *Hyperchol* hypercholesterolaemia, *CAG* coronary angiography, *ACEI* angiotensin-converting enzyme inhibitors, *ARB* angiotensin II receptor blockers. ^a^Nitrate vasodilators, beta blockers, or calcium antagonists. ^b^ACEI/ARB-inhibitors, beta blockers, calcium antagonists, or diuretics. ^c^Acetylsalicylic acid, dipyridamole, P2Y12 inhibitors, or vitamin K antagonists. *Sex difference *p* < 0.05

Sex differences were explored for certain risk factors in the presence of medication for angina relief, lowering blood pressure, statins, or antithrombotics, as depicted in Fig. 1. Women who reported chest pain had angina relief medication (75%) and blood-pressure-lowering medication (79%) more often than men with chest pain (60%, 63% respectively).

## Discussion

Based on the TWIST single-centre cohort study of patients with suspected INOCA, no significant sex differences were present in cardiac medication use. Previously observed sex differences were based on patients following myocardial infarction [11]. Patients in the TWIST study had no history of myocardial infarction, and subsequently the prevalence of cardiac medication use was lower overall. Herscovici and colleagues reviewed studies of patients with INOCA, showing large variability (2–59%) in hypertension/angina treatment and statin medication use [20]. In total 60% of the patients in the TWIST study with suspected INOCA received a combination of hypertension/angina therapy and statins, without sex differences. According to the 2019 ESC Guidelines pharmacological management of patients with stable CAD aims for relief of angina symptoms and to prevent the occurrence of cardiovascular events [4]. In total 62% of the patients received medication for angina relief, and 71% of the patients had either a blood-pressure-lowering medication, and antithrombotic medication, or a statin, whereas only 10% of the patients had none. Whether pharmacological management goals were met was not reported upon, which is a limitation. However, medication use in patients with a certain risk factor is depicted in Fig. 1, showing that blood-pressure-lowering medication was received by 93% of patients with hypertension, and statins in 81% of the patients with hypercholesterolaemia.

Though women and men differ in pharmacokinetics and pharmacodynamics, which can affect the suggested dosage, effectiveness, and side effects [12, 21], current guidelines have no sex-specific recommendations for cardiac medication use, which may be reflected in the absence of sex differences in the present study.

Experiencing consistent distress was related to more blood-pressure-lowering ACEI/ARB and diuretic use in women, and to angina-relief calcium antagonist use in men. Explorative analysis subsequently showed significantly more hypertension in distressed women (but not men) and more chest pain in distressed men (but not women), which is in line with observed higher blood-pressure-lowering and anti-anginal medication use. A meta-analysis has reported psychosocial stress to be related to a higher prevalence and an increased risk of hypertension [22]. This may partially reflect an adverse lifestyle in people experiencing psychosocial stress, but can also reflect having an increased allostatic load of biological stress mechanisms associated with the development of hypertension [23]. Similarly, depressive symptoms and anxiety have been found to be associated with chest pain in previously published findings in the TWIST cohort, though without sex differences [17]. In addition to examining sex differences in medication use it remains relevant to further unravel mechanisms relating psychological distress to cardiovascular disease processes.

In line with other studies, psychological distress, including anxiety and depressive symptoms, was higher in women compared to men [9, 24]. More psychotropic medication use is observed in women compared to men in European countries [25], which is partially reflected in more benzodiazepine use in women in the present study, but no higher prevalence of antidepressant use.

At 24 months, but not 12 months, men showed a lower adherence compared to women, and distressed men showed a lower adherence than non-distressed men at 12 months, but not at 24 months. Whether this generic aspect of self-care behaviour could mediate an adverse outcome in men in the long term remains to be examined.

There are a number of limitations. No information was available regarding sex-specific factors such as hormone replacement therapy or menopause status [26]. The term sex instead of gender was used, though gender effects cannot be excluded. Medication use was based on hospital records, which may not reflect current medication use. Adherence was self-reported as a single item, with a severely skewed distribution; thus a more extensive questionnaire could be more reliable. We did not record the change in medication use based on the CAG or CT diagnosis, but reported the medication use within a few weeks to months after the index CAG or CT scan. No information on dosage was reported, and no information on side effects was requested. The present study is a single-centre observational cohort study, and exploring medication use was a secondary analysis. Whether these findings translate to other (Dutch) cardiology practices remains to be examined.

## Conclusion

No sex differences were observed in cardiac medication use in patients with suspected INOCA, though consistent psychological distress in women reflected treatment for hypertension, and consistent distress in men reflected angina relief treatment.
